# Proprotein Convertase Subtilisin/Kexin Type 3 Promotes Adipose Tissue-Driven Macrophage Chemotaxis and Is Increased in Obesity

**DOI:** 10.1371/journal.pone.0070542

**Published:** 2013-08-06

**Authors:** Kai Kappert, Heike Meyborg, Jan Fritzsche, Daniel Urban, Janine Krüger, Ernst Wellnhofer, Ulrich Kintscher, Eckart Fleck, Philipp Stawowy

**Affiliations:** 1 Department of Medicine/Cardiology, Deutsches Herzzentrum Berlin, Berlin, Germany; 2 Institute of Laboratory Medicine, Clinical Chemistry and Pathobiochemistry, Center for Cardiovascular Research (CCR), Charité - Universitätsmedizin Berlin, Berlin, Germany; 3 Department of Translational Pharmacology, Center for Cardiovascular Research (CCR), Charité - Universitätsmedizin Berlin, Berlin, Germany; Wayne State University, United States of America

## Abstract

**Background:**

Matrix metalloproteinase (MMP)-dependent extracellular matrix (ECM) remodeling is a key feature in cardiometabolic syndrome-associated adipogenesis and atherosclerosis. Activation of membrane-tethered (MT) 1-MMP depends on furin (PCSK3). However, the regulation and function of the natural furin-inhibitor serpinB8 and thus furin/MT1-MMP-activity in obesity-related tissue inflammation/remodeling is unknown. Here we aimed to determine the role of serpinB8/furin in obesity-associated chronic inflammation.

**Methods and Results:**

Monocyte → macrophage transformation was characterized by decreases in serpinB8 and increases in furin/MT1-MMP. Rescue of serpinB8 by protein overexpression inhibited furin-dependent pro-MT1-MMP activation in macrophages, supporting its role as a furin-inhibitor. Obese white adipose tissue-facilitated macrophage migration was inhibited by furin- and MMP-inhibition, stressing the importance of the furin-MMP axis in fat tissue inflammation/remodeling. Monocytes from obese patients (body mass index (BMI) >30kg/m^2^) had higher furin, MT1-MMP, and resistin gene expression compared to normal weight individuals (BMI<25kg/m^2^) with significant correlations of BMI/furin and furin/MT1-MMP. *In vitro*, the adipocytokine resistin induced furin and MT1-MMP in mononuclear cells (MNCs), while MCP-1 had no effect.

**Conclusions:**

Acquisition of the inflammatory macrophage phenotype is characterized by an imbalance in serpinB8/furin, leading to MT1-MMP activation, thereby enhancing migration. Increases in MT1-MMP and furin are present in MNCs from obese patients. Dissecting the regulation of furin and its inhibitor serpinB8 should facilitate targeting inflammation/remodeling in cardiometabolic diseases.

## Introduction

Chronic inflammation of adipose tissue is a key process in the development and progression of insulin resistance and atherosclerosis, significantly contributing to obesity-associated increases in cardiovascular mobidity and mortality [1,2]. A characteristic of this chronic low-grade inflammatory process is the invasion of white adipose tissue (WAT) by mononuclear cells (MNCs), and their subsequent interaction with adipocytes. Consequently, elevated levels of cytokines/adipokines are released, further facilitating the inflammatory process [3].

This cardiometabolic syndrome-associated tissue inflammation/remodeling typically requires the expression of matrix metalloproteinases (MMPs) [4], present with their counterpart TIMPs (tissue inhibitors of matrix metalloproteinases) in WAT of obese animal models [5]. Recently, gene polymorphisms in membrane-tethered (MT) 1-MMP (MMP-14) have been linked to human obesity and insulin-resistance characteristics, and knockout of this protease was shown to protect from high-fat diet induced adipose tissue remodeling/expansion in mice [6].

MT1-MMP gains activation intracellularly, followed by anchoring of the active protease to the cell membrane, whereas other MMPs (e.g. MMP-2, -9) are typically expelled as zymogens and subsequently activated in the interstitium [7]. Pro-MT1-MMP activation depends on prosegment cleavage at RRKR^111^ ↓Y^112^, involving the proprotein convertase subtilisin/kexin type 3 (PCSK3) known as furin [8]. For simplification, the nine mammalian PCSKs identified so far may be separated according to their cleavage preferences: seven of them activate precursors at di-basic amino acid residues (consensus motif: K/R-*X*n-K/R↓, where n= 0, 2, 4 or 6 and *X* is any amino acid), including PCSK1, PCSK2, PCSK3, PCSK4, PCSK5 (also named PC5/6), PCSK6 (also named PACE4) and PCSK7 (also named PC3) [9]. The other two PCSKs prefer single or pairs of hydrophobic amino acids for cleavage and are named SKI-1/PCSK8 and PCSK9, both identified as key regulators in lipid metabolism [9]. However, despite having common cleavage preferences, PCSK selectivity/specificity is additionally determined by cellular/intracellular expression profiles, differences in regulation in diseases and unique structural/biochemical requirements in substrates [9]. Thus, PCSK1 and PCSK2 are typically found in secretory granules of neuroendocrine cells and PCSK4 is confirmed to germinal cells, whereas others like furin or PCSK5 are located in the constitutive pathway, cycling between the trans-Golgi-network (TGN) and cell membrane via endosomal pathways [9]. Previously, we demonstrated the upregulation of furin and PCSK5 in monocyte → macrophage transformation, enabling MNCs to participate in tissue inflammation [10]. Whereas furin-specific siRNA significantly inhibited MT1-MMP activity in macrophages, a PCSK-inhibitor completely abrogated pro-MT1-MMP activation, supporting the notion that both furin and PCSK5 target pro-MT1-MMP along its maturation pathway [10]. Indeed, based on their overlap in expression and substrate specificity, these two PCSKs are commonly regarded as “furin-like” PCSKs [11].

However, the expression and impact of furin-like PCSKs and thus MT1-MMP activation in obesity-related WAT inflammation in cardiometabolic syndrome still have to be elucidated. In addition, little is known about the (patho) physiological regulation of the naturally occurring furin-inhibitor serpinB8, demonstrated to form an SDS-stable complex with its substrate furin [12]. SerpinB8 belongs to the ovalbumin-like serine protease inhibitor family and was found to be restricted to neuroendocrine cells, squamous epithelial cells, platelets and MNCs [12-15].

Thus, the present study investigated the regulation of serpinB8 in macrophage maturation, subsequent furin-dependent MT1-MMP activation, and consecutive inflammatory MNC-driven adipose tissue-invasion. Moreover, we examined the regulation of furin, serpinB8 and MT1-MMP in an animal model of obesity as well as in obese patients.

## Methods

### Ethics statement

Human data: Clinical data and monocyte mRNA were derived from patients recruited for the trial registered as ClinicalTrials.gov-ID NCT00561327. The study was approved by the ethics committee of the Charité - Universitätsmedizin Berlin, Germany. All patients provided written informed consent.

Animal data: This study was carried out in strict accordance with the recommendations in the Guide for the Care and Use of Laboratory Animals of the Landesamt für Gesundheit und Soziales (LAGeSo) – Berlin, Germany. The protocol was approved by the Committee of the LAGeSo (Permit Number: T0007/11).

### Materials

Cell culture media and materials were purchased from Invitrogen. The furin-like PCSK inhibitor decanoyl-RVKR-chloromethylketone (dec-CMK) and the fluorogenic furin-like PCSK substrate pyr-RTKR-AMC were purchased from Bachem; the broad spectrum hydroxamate class MMP-inhibitor GM6001 (Ilomastat) was from Chemicon. Recombinant serpinB8 protein was from Abnova, human MCP-1 was purchased from Millipore, and human resistin was from PeproTech. Recombinant TIMP-1, -2 and -3 proteins, gelatin, phorbol 12-myristate 13-acetate (PMA) and brefeldin-A (BFA) were purchased from Sigma. The following antibodies were used: monoclonal anti-furin MON-152 (Enzo Life Science), anti-actin (Sigma) and anti-serpinB8 (R&D Systems). The MT1-MMP antibody directed against the hinge region (AB815), anti-TIMP-1, anti-TIMP-2 and anti-TIMP-3 were from Chemicon. Antibodies against HSP-90, canexin and PARP-1 were from Amersham.

### Cell Culture

The human monocytic THP-1 cell line was purchased from Deutsche Sammlung von Mikroorganismen und Zellkulturen (DSMZ), Braunschweig, Germany, and cultured in RPMI 1640 (containing 10% FCS with 2 mmol/L L-glutamine, 100 U/mL penicillin and 100 µg/mL streptomycin) at 95% relative humidity and 5% CO_2_ at 37^°^ C. Differentiation of THP-1 monocytes to macrophages (THP/ϕ) occurred in the presence of 100 nmol/L PMA (48h). Following differentiation to THP/ϕ, cells were washed, PMA removed and further experiments were done in serum-free medium. Pretreatment of cells was performed as indicated in the figure legends. For serpinB8 protein transfection the ProteoJuice Protein Transfection reagent (Novagen) was used as recommended by the manufacturer. All experiments were done n≥3.

### Immunoblotting

Briefly, cells were lysed in buffer (20 mmol/L Tris, pH 7.5, 150 mmol/L NaCl, 1 mmol/L EDTA, 1 mmol/L EGTA, 1% Triton X-100, 2.5 mmol/L Na-pyrophosphate, 1 mmol/L Na _3_VO_4_) containing freshly dissolved protease inhibitors (Complete EDTA-free, Boehringer). For subcellular protein expression analyses in THP-1 monocytes and THP/ϕ, the ProteoExtract Subcellular Proteome Extraction Kit (Calbiochem) was used according to the manufacturers’ instructions. Up to 50 µg of proteins were subjected to 10% reducing SDS-PAGE. Following standard blotting procedures and exposure to primary antibodies, HRP-conjugated anti-mouse and anti-rabbit secondary antibodies (1:25,000) were used with enhanced chemiluminescence (Amersham) for visualization.

### Enzyme activity assay

Furin-like PCSK activity was determined using 10 µg protein in 10 µmol/L assay buffer (100 mmol/L Hepes (pH 7.5), 0.5% Triton X-100, 1 mmol/L CaCl2, 1 mmol/L 2-mercaptoethanol) and 0.5 µmol/L internally quenched fluorescent furin-substrate pyr-RTKR-AMC at 37° C for 1h. Furin activity was quantified by the release of the fluorescent AMC moiety, measured with a fluorospectrometer (Fluostar OPTIMA, 320 nm excitation/425 nm emission).

### Patients’ characteristics

Hypertensive patients (n=26) were recruited in the Department of Medicine/Cardiology at the Deutsche Herzzentrum Berlin, Germany. Patients were divided into three BMI categories (mass (kg) / height (m)^2^) based on conventional definitions: normal weight (<25 kg/m^2^), n=5; overweight (25-30 kg/m^2^), n=7; obese (>30 kg/m^2^), n=14. Patients’ characteristics, diagnoses, and medication are summarized in Tables 1 and 2. In the obese group, gamma-GT was only documented for n=12; LDH, sodium and blood pressure for n=13.

**Table 1:** Characteristics of the Study Population, diagnoses (mean ± S.D.).

		**BMI**							
**Patients’ Characteristics**		< 25 kg/m^2^ n=5		25-30 kg/m^2^ n=7		> 30 kg/m^2^ n=14		*P* for difference (< 25 kg/m^2^ vs. > 30 kg/m^2^)	
Sex, *male/female*		4/1		7/0		11/3		n.s.	
Age, *years*		62.2±6.5		64.1±9.8		65.0±7.8		n.s.	
Body Mass Index, *kg/m* ^2^		24.3±0.6		27.9±1.0		32.7±2.1		<0.001*	
Weight, *kg*		73.2±3.3		87.1±9.0		96.9±10.3		<0.001^†^	
Height, *cm*		172.2±2.9		176.4±7.9		171.9±6.6		n.s.	
Systolic blood pressure, *mmHg*		128.2±26.1		135.1±11.5		155.5±14.6		n.s.	
Diastolic blood pressure, *mmHg*		74.4±10.7		75.3±11.9		84.1±7.7		n.s.	
Heart Rate, *min*		67.0±9.1		68.0±12.5		71.4±10.7		n.s.	
		**BMI**							
**Diagnosis**		< 25 kg/m^2^ n=5 (%)		25-30 kg/m^2^ n=7 (%)		> 30 kg/m^2^ n=14 (%)		*P* for difference	
Hypertension		5 (100%)		7 (100%)		14 (100%)		n.s.	
Diabetes		1 (20%)		1 (14%)		9 (64%)		n.s.	
Coronary Artery Disease		3 (60%)		6 (86%)		11 (79%)		n.s.	

* < 25 kg/m^2^ vs. 25-30 kg/m^2^, p<0.001; 25-30 kg/m^2^ vs. > 30 kg/m^2^, p<0.001; < 25 kg/m^2^ vs. > 30 kg/m^2^, p<0.001

^†^ < 25 kg/m^2^ vs. 25-30 kg/m^2^, p=0.026; 25-30 kg/m^2^ vs. > 30 kg/m^2^, p=0.159; < 25 kg/m^2^ vs. > 30 kg/m^2^, p<0.001

**Table 2:** Characteristics of the Study Population, medication, n (%).

	**BMI**			
**Medication**	< 25 kg/m^2^ n=5 (%)	25-30 kg/m^2^ n=7 (%)	> 30 kg/m^2^ n=14 (%)	*P* for difference
Renin-Angiotensin-Blockers	3 (60%)	5 (71%)	13 (93%)	n.s.
Statins	3 (60%)	6 (86%)	10 (71%)	n.s.
Antiplatelets	4 (80%)	6 (86%)	12 (86%)	n.s.
Diuretics	3 (60%)	3 (43%)	11 (79%)	n.s.
Calcium Channel Blockers	3 (60%)	3 (43%)	7 (50%)	n.s.
Beta-Blockers	4 (80%)	4 (57%)	11 (79%)	n.s.

### Blood sample preparation/storage and serum parameters

From all patients 40 mL venous blood was withdrawn from the antecubital vein for monocyte isolation. Besides isolation of monocytes, serum was separated and parameters analyzed at the routine laboratory at the Deutsche Herzzentrum Berlin, Germany (Table 3).

**Table 3:** Characteristics of the Study Population, serum parameters, (mean ± S.D.).

	**BMI**			
**Serum parameters**	< 25 kg/m^2^ n=5	25-30 kg/m^2^ n=7	> 30 kg/m^2^ n=14	*P* for difference
Glucose, *mg/dL*	104.8±19.6	94.9±19.0	122.8±43.4	n.s.
Creatinine, *mg/dL*	1.19±0.31	1.13±0.20	1.12±0.21	n.s.
Sodium, *mmol/L*	141.1±1.8	141.9±1.1	138.6±4.5	n.s.
Potassium, *mmol/L*	3.80±0.23	3.85±0.39	3.83±0.50	n.s.
γGT, *U/L*	125.2±141.0	39.0±17.7	72.2±54.6	n.s.
GOT, *U/L*	31.8±8.7	30.0±7.0	39.7±38.5	n.s.
LDH, *U/L*	197.6±30.9	191.7±34.8	212.5±82.7	n.s.
Cholesterol, *mg/dL*	168.6±49.9	164.3±41.9	181.4±47.9	n.s.
LDL-Cholesterol, *mg/dL*	105.2±38.9	94.3±36.8	110.4±44.1	n.s.
HDL-Cholesterol, *mg/dL*	40.8±9.9	43.3±6.4	48.5±13.1	n.s.
Triglycerides, *mg/dL*	137.6±54.0	191.7±74.5	147.0±83.3	n.s.

### Human monocyte isolation

The Ficoll Hypaque monocyte isolation method employed a liquid density gradient medium of Ficoll 400 and sodium metrizoate or sodium diatrizoate solution. Vacutainer Cell Preparation Tubes (Becton Dickinson) were filled with peripheral whole blood and centrifuged to isolate the MNCs above the medium. For separation of monocytes from other MNCs the Dynal monocyte negative isolation kit was applied (Invitrogen). Non-monocytes were depleted by magnetic Dynabeads after preincubation with an antibody mix against non-monocytes. Finally, bead- and antibody-free pelleted monocytes were either shock-frozen in liquid nitrogen and stored at -80° C, or were grown in RPMI 1640 (containing 10% FCS, 2 mmol/L L-glutamine, 100 U/mL penicillin and 100 µg/mL streptomycin) at 95% relative humidity and 5% CO_2_ at 37^°^ C. Culture-induced macrophage differentiation occurred at ~ day 5 and was evident by cell morphology and the expression of vimentin.

### Chemotaxis

THP-1 macrophage transwell chemotaxis was analyzed using transwell cell culture chambers with gelatin-coated (0.2%) polycarbonate membranes (8 µm pores) (Becton Dickinson). In inhibition experiments cells were pretreated with inhibitors or transfected with serpinB8 protein as indicated. The number of cells per high power field (HPF, magnification x320) that had migrated to the lower surface of the filters after 4h was determined microscopically. Four randomly chosen HPFs were counted per filter. Experiments were performed in triplicates and were repeated at least three times.

### RNA isolation and quantitative reverse transcriptase real-time polymerase chain reaction (qRT-PCR)

RNA from frozen monocyte pellets, cultured THP-1 monocytes/macrophages or from mouse adipose tissue was isolated using an RNAeasy Mini or Micro kit from Qiagen (Hilden, Germany). RNA was transcribed to complementary DNA (cDNA) with random primers (Promega, Madison, WI, USA), and SuperScript II Reverse Transcriptase (Invitrogen GmbH, Karlsruhe, Germany) on a thermocycler (Biometra, Göttingen, Germany). cDNA was subjected to qRT-PCR using the Power SYBRGreen PCR Master Mix Reagent Kit (Applied Biosystems, Foster City, CA, USA). All primers were used at 100 nmol/L. Sequences are listed in the Supporting Table S1. The reaction was performed in duplicate to quadruplicate with an *Mx3000P® QPCR System* (Stratagene, La Jolla, CA, USA). Amplification reaction data were analyzed by the complementary Mx3000P analysis software. Target gene expression was normalized to the average expression of the 18S ribosomal RNA housekeeping gene.

### Animals and adipose tissue culture

Male wild-type and ob*/*ob C57Black/6 mice were purchased from Janvier (St.-Berthevin Cedex, France). Animals had access to regular chow diet and water *ad libitum*, and were kept at 12h/12h dark/light conditions. Animals aged 11 weeks were used for the experiments. After body weight measurement, animals were euthanized under deep isoflurane-anesthesia by rapid cervical dislocation. Gonadal WAT and subcutaneous fat was isolated and weights determined, tissues were shock-frozen in liquid nitrogen and stored at -80° C until further use for gene expression analyses. Additionally, pieces of 1g WAT were mechanically minced and cultured for 24h in 1 mL DMEM without serum at 37° C [16]. Thereafter conditioned media was stored at -20° C until use for chemotaxis assays. Characteristics of animals are given in Supporting Table S2.

### Statistical Methods

Results of the gene expression analyses and laboratory serum parameters are reported as mean ± standard deviation (SD) or mean ± standard error of the mean (SEM), as indicated in the tables and figure legends. Differences were analyzed by ANOVA followed by least significance difference post-hoc test, or *t*-test, as appropriate. Patients’ characteristics and diagnoses were analyzed by Oneway ANOVA with or without post-hoc Tamhane test, respectively. A *p*-value <0.05 was regarded as significant.

## Results

### Monocyte transformation leads to a decline of serpinB8 and subsequent pro-MT1-MMP activation

**Figure 1:**
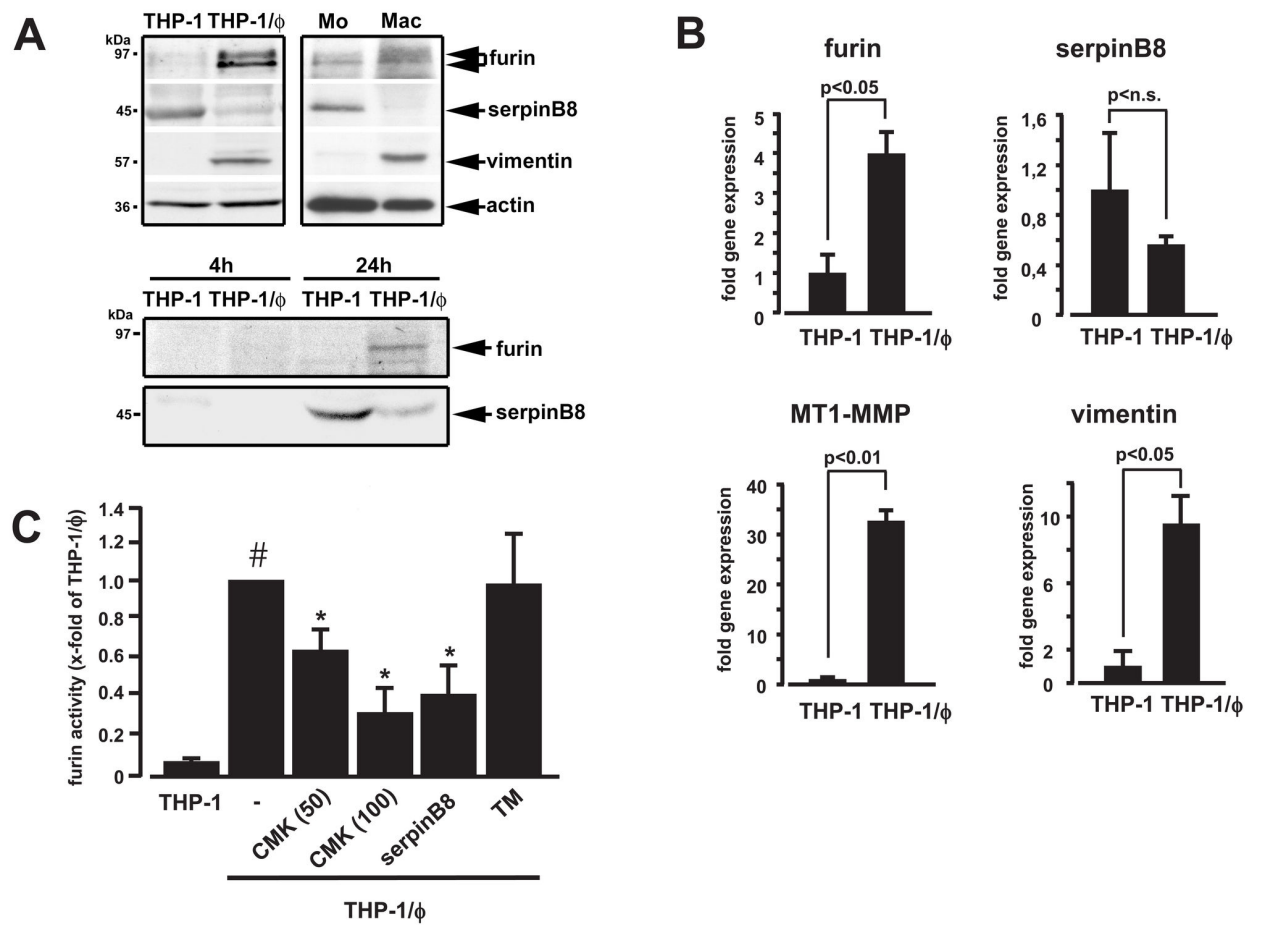
Regulation of furin and serpinB8 during monocyte transformation. (**A**) Monocyte transformation (THP-1 cells (*upper left*), primary monocytes (*upper right*)) to macrophages (THP-1/ϕ) was evident by vimentin expression. Furin was increased and serpinB8 was decreased in macrophages (*upper panel*). Furin was detected in its pro- and active form (actin-reblotting for protein loading). Immunoblotting of supernatants demonstrated that furin was time-dependently shed from THP-1/ϕ, whereas serpinB8 was primarily released from monocytic cells (both at 24h; *lower panel*). (**B**) QRT-PCR demonstrated significant upregulation of furin and MT1-MMP accompanying vimentin in THP-1/ϕ, whereas decreases in serpinB8 were non-significant. (**C**) Cell-transformation increased furin-like PCSK activity (#p<0.05 THP-1/ϕ vs. THP-1 cells), which was concentration-dependently inhibited by dec-CMK (CMK, 50 and 100 µmol/L; 12h), or transfection of THP-1/ϕ with serpinB8 protein (5 µg/mL; 12h). TM = transfection medium. *p<0.05 vs. THP-1/ϕ. n=3.

Regulation of serpinB8/furin in monocyte → macrophage transformation was investigated using human primary monocytes and monocytic THP-1 cells. Immunoblotting revealed opposing regulation of furin and serpinB8 in both cell types, with strong increases in furin and simultaneous decreases of serpinB8 upon transformation to the inflammatory macrophage phenotype (Figure 1A, upper panel). Next we examined whether serpinB8 losses were due to release from cells. Immunoblotting of conditioned media demonstrated significant amounts of serpinB8 being time-dependently released only from monocytic cells, whereas furin was primarily shed from macrophages (Figure 1A, lower panel). The imbalance in the furin/serpinB8 ratio was confirmed using qRT-PCR, demonstrating significant increases of furin mRNA in macrophages compared to monocytes, with serpinB8 transcripts only slightly decreasing in macrophages (Figure 1B). Additionally, MT1-MMP mRNA was strongly induced in macrophages, along with vimentin [10], validating monocyte transformation (Figure 1B). To further examine the capacity of serpinB8 as furin-inhibitor, specific enzyme activity assays were performed using macrophages transfected with serpinB8 protein. Furin-like PCSK enzymatic activity was strongly increased in macrophages compared to monocytes. This enhanced activity was significantly inhibited by the specific synthetic furin-like PCSK inhibitor dec-CMK [17], and by serpinB8 transfection (Figure 1C).

**Figure 2:**
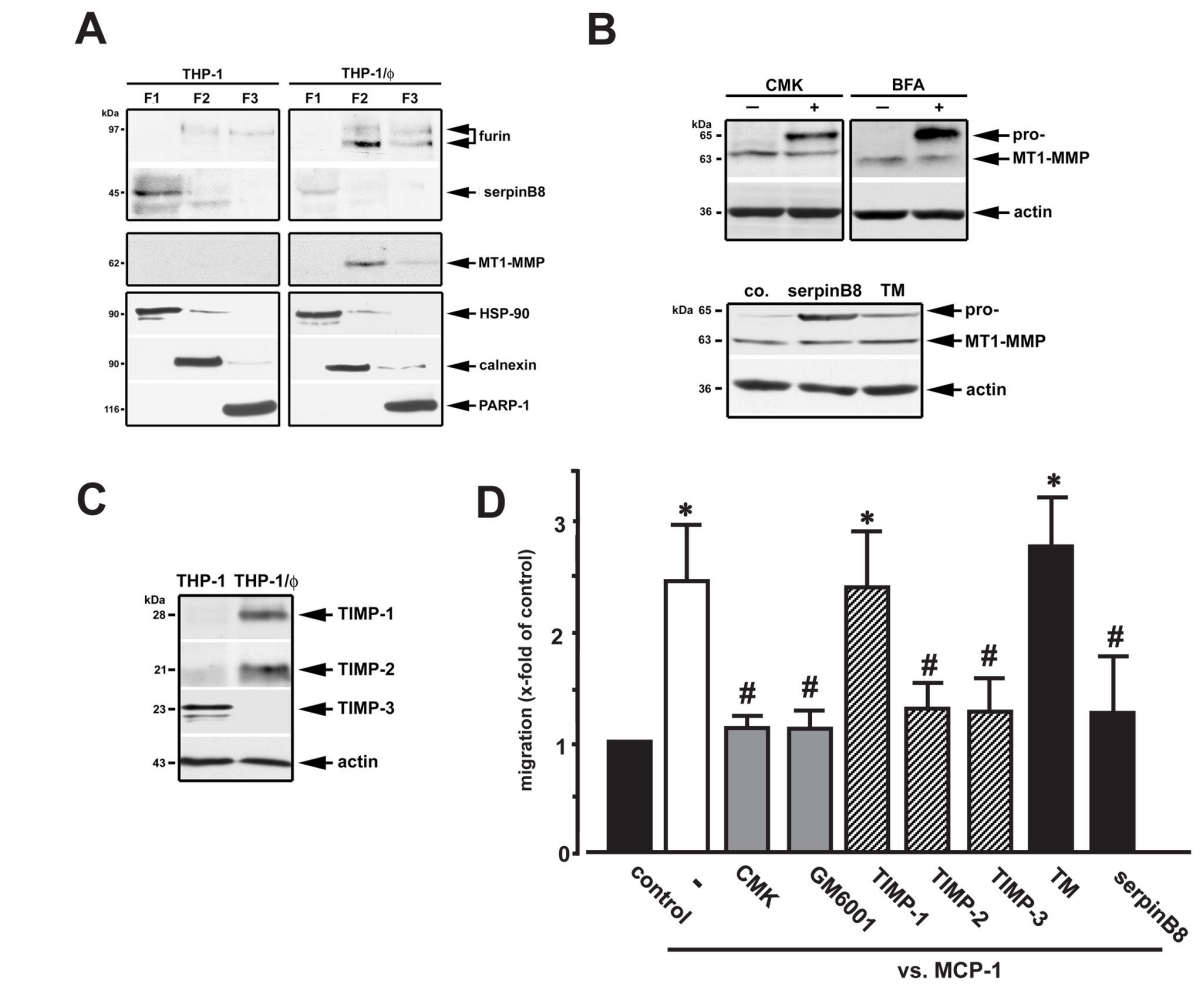
Subcellular localization and interaction of furin, MT1-MMP and serpinB8. (**A**) Subcellular localization of furin, serpinB8 and MT1-MMP was compared in THP-1 and THP-1/ϕ using cell fractionation. SerpinB8 was mostly found in THP-1 cells in the cytosolic fraction (F1). Furin and MT1-MMP were highly expressed in THP-1/ϕ in the organelle fraction (F2), and to a lesser extent in the membrane fraction (F3). (**B**) Dec-CMK (CMK, 50 µmol/L; 12h) or BFA (10 µg/mL; 30 min) inhibited pro-MT1-MMP activation in THP-1/ϕ, evident by increases in its 65 kDa pro-form (upper panel). THP-1/ϕ were transfected with serpinB8 (5 µg/mL; 12h) or treated with transfection medium (TM) alone, and further processed for immunoblotting. SerpinB8 inhibited pro-MT1-MMP activation, evident by increases in the pro-form of MT1-MMP (*lower panel*). (**C**) Monocyte (THP-1)/macrophage (THP-1/ϕ) transformation was accompanied by increases of TIMP-1 and TIMP-2, whereas TIMP-3 was downregulated (actin-reblotting for protein loading). (**D**) Macrophage chemotaxis towards MCP-1 (10 ng/mL) was inhibited by the furin-like PCSK inhibitor dec-CMK (CMK, 50 µmol/L), serpinB8 (5 µg/mL), and the MMP-inhibitors GM6001 (50 µmol/L), TIMP-2 and TIMP-3 (both 200 ng/mL). TIMP-1 (200 ng/mL) and transfection medium (TM) had no effect (*p<0.05 vs. controls, co.; #p<0.05 vs. MCP-1 alone). n=3.

Next we explored the interplay of furin/serpinB8 within the MT1-MMP-activation network using cell fractionation to dissect subcellular localizations (Figure 2A). In monocytes, serpinB8 was mostly present in the cytosol (F1 fraction; HSP-90 positive), with lesser amounts in the organelle fraction (F2; calnexin positive). Furin and MT1-MMP were strongly increased in macrophages, emphasized by subcellular localization in the organelle fraction (F2), with some furin also detectable in the membrane fraction (F3; PARP-1 positive). In contrast, serpinB8 was virtually absent in these cells. Since furin and MT1-MMP cycle between the TGN and the cell surface [11], the finding of a cytoplasmic localization of serpinB8 in monocytes and its downregulation in macrophages does not *per se* exclude its function as furin-inhibitor. Thus, the impact of serpinB8 transfection on MT1-MMP activation in macrophages was compared to the PCSK inhibitor dec-CMK, as well as to brefeldin A (BFA). BFA causes a microtubule-mediated fusion of the trans-Golgi network (TGN) and early endosomes, dissecting it structurally and functionally from the Golgi-complex [18,19]. Immunoblotting demonstrated that both dec-CMK and BFA significantly reduced MT1-MMP activation, evident by increases in its 65 kDa pro-form. This confirms furin-like PCSKs as major pro-MT1-MMP convertases in macrophages, and demonstrates that MT1-MMP activation by furin-like PCSKs occurs in the TGN/post-TGN compartments where precursor protein activation by di-basic PCSKs localized in the constitutive pathway typically occurs (Figure 2B, upper panel). Along this line, serpinB8 protein transfection resulted in a significant inhibition of pro-MT1-MMP activation as well (Figure 2B, lower panel).

### Furin-driven pro-MT1-MMP activation is crucial for adipose tissue inflammation

Typically and concomitant to MMP activation is a release of TIMPs. Within the TIMP family TIMP-2 is unique, because it not only inhibits MMPs, but is also obligatory in a cell surface-associated MT1-MMP/pro-MMP-2/TIMP-2 complex, facilitating pro-MMP-2 activation [7].

Associated with furin/MT1-MMP upregulation in macrophages, increases in TIMP-1 and TIMP-2 were detected, whereas TIMP-3 was lost upon monocyte transformation (Figure 2C). TIMP-1 is a potent inhibitor of MMP-9, whereas TIMP-2 and -3 are capable of inhibiting MT1-MMP [20]. Thus, chemotaxis experiments with TIMPs, synthetic inhibitors of MMPs and furin, as well as serpinB8 transfection were used to investigate the control of macrophage motility. MCP-1, a key regulator of adipose tissue macrophage recruitment, was used as chemoattractant [16]. MCP-1-directed migration was significantly inhibited by either furin-like PCSK inhibition with dec-CMK or serpinB8, as well as by MMP inhibition (with GM6001) or an excess of exogenous TIMP-2 or TIMP-3 (Figure 2D). In contrast, addition of TIMP-1 had no impact on the increase of MCP-1-directed macrophage chemotaxis. These results support the concept that macrophage migration requires a serpinB8-controlled furin-dependent MMP activation cascade.

**Figure 3:**
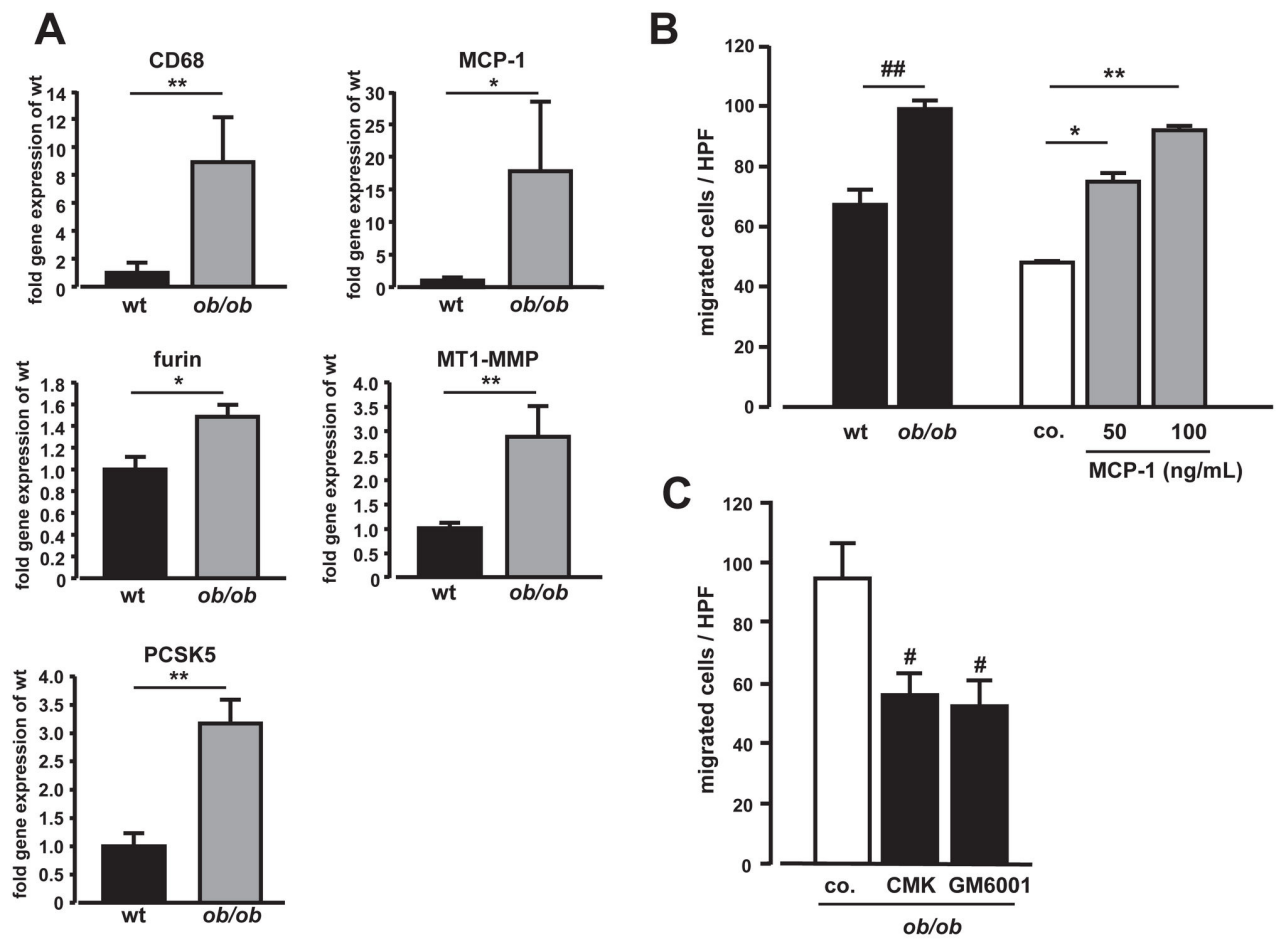
Obese white adipose tissue (WAT) gene expression and impact on macrophage migration. (**A**) WAT derived from wild-type (wt) and obese ob*/*ob mice was subjected to qRT-PCR. Increased gene expression of CD68, MCP-1, furin, MT1-MMP, and PCSK5 was found in ob*/*ob mice (*p<0.05; **p<0.01 vs. wt). (**B**) Supernatants from WAT cultures of ob*/*ob mice increased THP-1/ϕ migration (^##^p<0.05 vs. wt), comparable to MCP-1 (*p<0.05 vs. control, co.; **p<0.01 vs. 50 ng/mL MCP-1). (**C**) Supernatant-facilitated migration was inhibited by dec-CMK (CMK, 50 µmol/L; 12h) or the MMP-inhibitor GM6001 (50 µmol/L; 12h) (both #p<0.05 vs. controls, co.). n=3.

The importance of this proteolytic cascade for a low-grade inflammatory state, present in obesity, was further investigated in ob*/*ob mice (Supporting Table S2). WAT from ob*/*ob mice was characterized by inflammation, evident by a significant upregulation of CD68 and MCP-1 gene expression compared to wild-type mice (Figure 3A). Moreover, transcript levels of furin and MT1-MMP were significantly upregulated in inflamed adipose tissue. In addition, PCSK5, which may substitute for furin due to overlapping substrate specificity and subcellular localization [9], was highly increased (Figure 3A). To further investigate WAT-driven macrophage infiltration, supernatants from wild-type or ob*/*ob mice adipose tissue cultures were used as chemoattractants. Comparable to the enhanced migration induced by MCP-1, adipose tissue supernatants from ob*/*ob mice strongly facilitated macrophage motility (Figure 3B). This was inhibited by both furin- and MMP-inhibition, supporting the concept that furin-initiated MMP-activation mediates adipose tissue-driven macrophage invasion (Figure 3C).

### Furin and MT1-MMP are increased in monocytes from obese hypertensive patients

**Figure 4:**
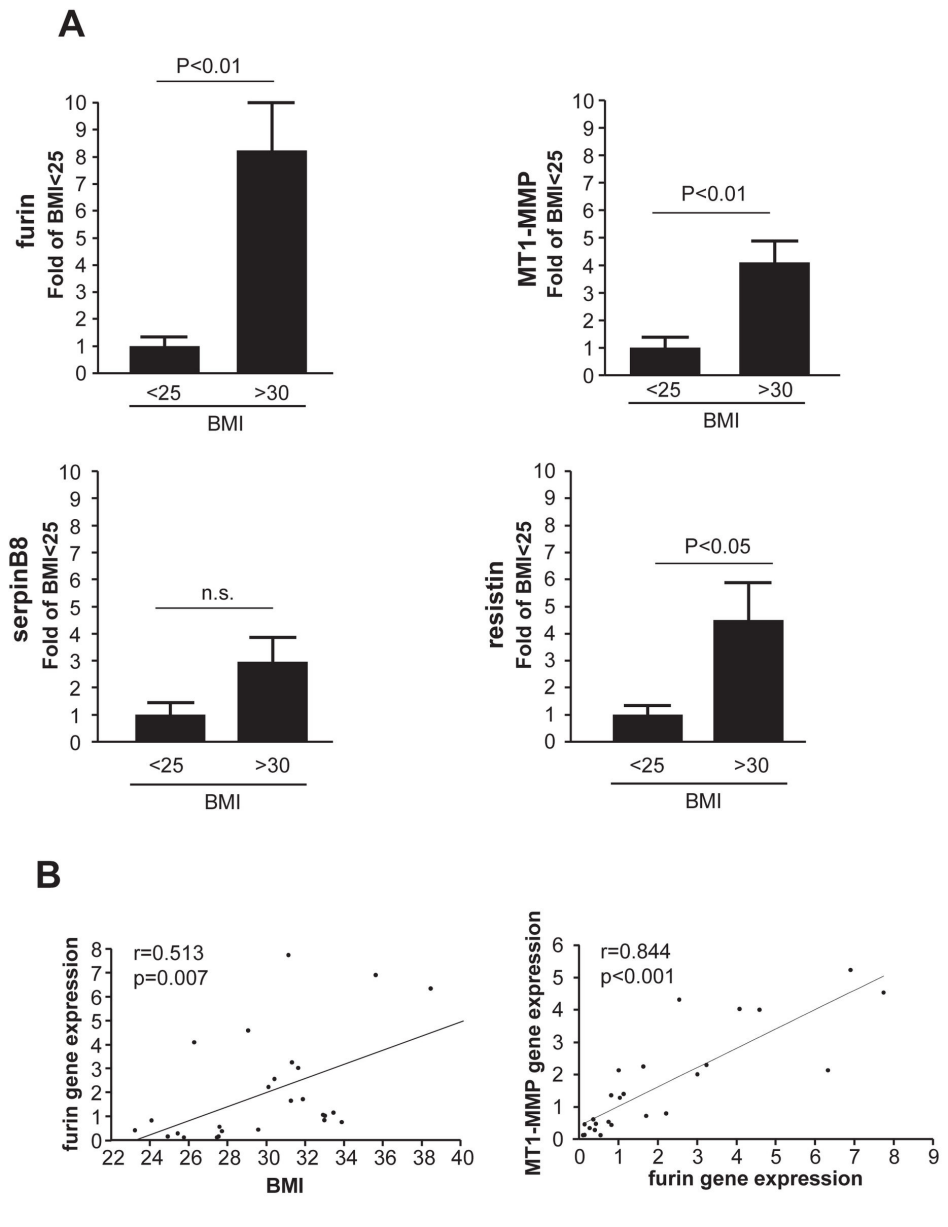
BMI-based gene expression in monocytes. (**A**) Monocytes isolated from patients were subjected to qRT-PCR. Furin, MT1-MMP, and resistin gene expression was higher in patients with a BMI>30 kg/m^2^ compared to patients with a BMI<25 kg/m^2^, whereas higher serpinB8 transcript levels did not reach statistical significance. (**B**) Furin levels correlated with BMI (*left*) and MT1-MMP (*right*) gene expression.

The expression of furin, its inhibitor serpinB8, and MT1-MMP was investigated in monocytes derived from obese patients. In addition, resistin, known to be increased in obese patients, was determined [21]. Detailed patients’ characteristics, serum parameters, and medications are presented in Tables 1, 2 and 3. Significant differences between groups based on BMI were found for systolic blood pressure, but not for sex, age, height, diastolic blood pressure, heart rate, diagnosis, concomitant medication, or analyzed serum parameters (Tables 1-3). Cardiovascular risk increases with the BMI [1], and further comparisons were focused on groups of BMI<25 kg/m^2^ and BMI>30 kg/m^2^. Compared to normal weight patients (BMI<25 kg/m^2^), significant increases in transcript levels were detected for furin, MT1-MMP and resistin in monocytes isolated from obese patients with BMI>30 kg/m^2^ (Figure 4A), while serpinB8 did not significantly differ. To support and substantiate the findings of parallel increases of furin and MT1-MMP, correlation analyses were performed. To this end, furin gene expression significantly correlated with BMI, and gene expression of MT1-MMP correlated with its convertase furin (Figure 4B).

### Resistin regulates a furin - MT1-MMP activation axis in monocytic cells

**Figure 5:**
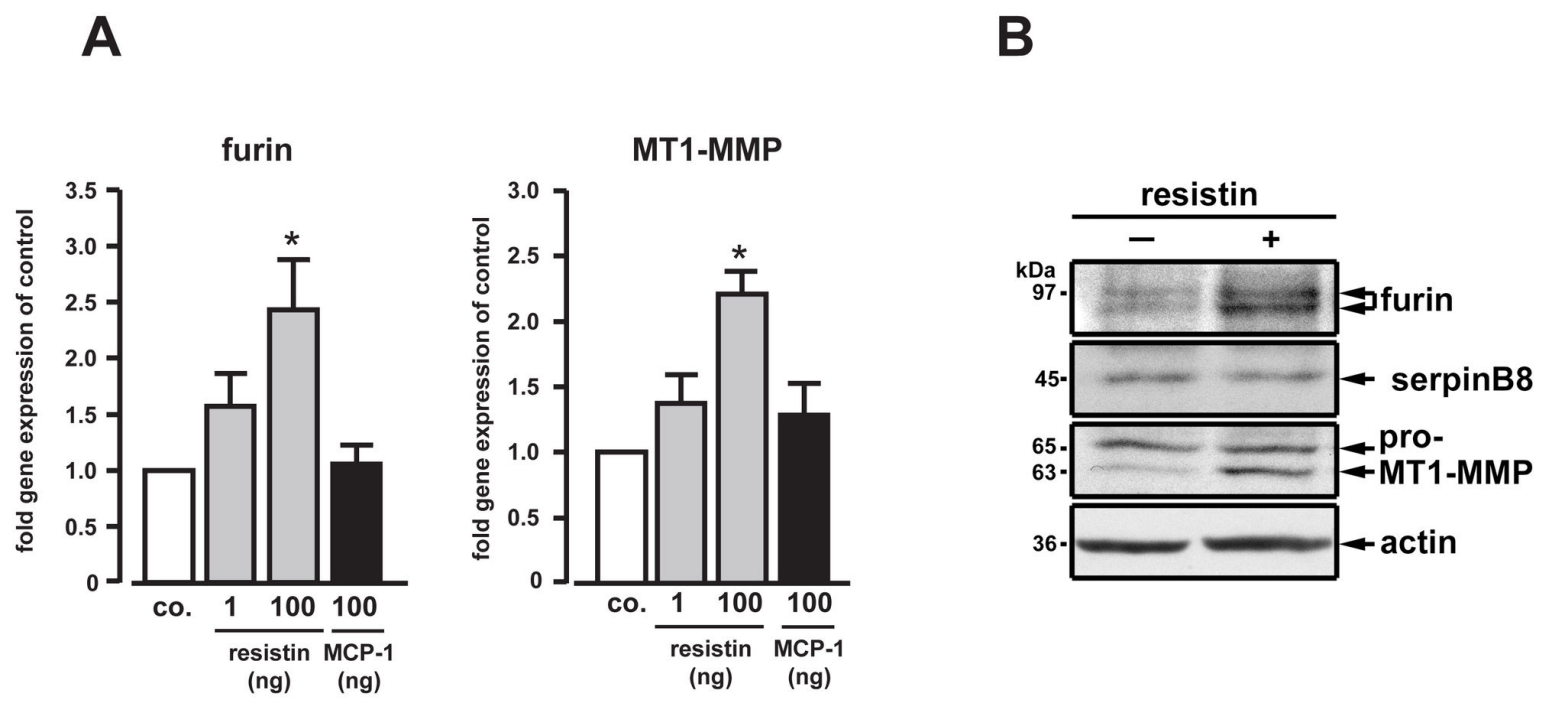
Impact of resistin on monocyte furin, MT1-MMP and serpinB8 expression. (**A**) THP-1 monocytes were stimulated with resistin or MCP-1 and subjected to qRT-PCR. Resistin increased furin and MT1-MMP, whereas MCP-1 had no effect (*p<0.05 vs. control, co.). (**B**) Immunoblotting revealed that resistin (100 ng/mL) increased furin and MT1-MMP. In contrast, protein levels of serpinB8 were not affected (actin-reblotting for protein loading). n=3.

We found synchronized increases in MCP-1, furin, PCSK5 and MT1-MMP transcripts in WAT from ob*/*ob mice, and resistin gene expression paralleling increases in furin and MT1-MMP in monocytes from patients. Thus, the impact of these two adipokines on furin and MT1-MMP levels in MNCs was investigated further. QRT-PCR revealed a concentration-dependent increase of furin and MT1-MMP upon resistin stimulation, whereas MCP-1 had no effect (Figure 5A). Immunoblotting confirmed that increases in furin and MT1-MMP mRNAs were accompanied by protein upregulation, whereas serpinB8 was not affected by resistin (Figure 5B). Thus, resistin contributes to an inflammatory phenotype involving the regulation of a furin/MT1-MMP-axis.

## Discussion

This study dissected the impact and interplay of the PCSK family member furin (PCSK3) and its inhibitor serpinB8 in WAT inflammation and obesity. We demonstrate that acquisition of the invasive macrophage phenotype is driven by attenuation of serpinB8 and upregulation of furin, resulting in increased furin-like PCSK activity. This leads to furin-dependent activation of pro-MT1-MMP, which is crucial for obese WAT-driven macrophage chemotaxis. Furthermore, we show that furin and MT1-MMP are significantly increased in obese patients and that furin is upregulated by the adipocytokine resistin in monocytes *in vitro*, supporting the concept that PCSKs contribute to obesity-associated chronic low-grade inflammation.

WAT inflammation is characterized by macrophage invasion, recruited by chemokines like MCP-1 [16,22]. Here we demonstrate that furin-like PCSKs are crucial for MCP-1-directed macrophage migration, since both the cell permeable peptide-based irreversable furin-like PCSK inhibitor dec-CMK as well as transfection of serpinB8 significantly inhibited migration. SerpinB8, the naturally occurring furin-inhibitor, belongs to the serine protease inhibitor family, comprising proteins with diverse functions but comparable structure, in which *in vivo* specificity is fine tuned by specific amino acid residues in reactive sites, exosite and cofactor necessities [14,23]. Knowledge about the regulation of serpinB8 in diseases is sparse. Alterations in its expression have been described during matrix remodeling following kidney injury [24]. In platelets serpinB8 is released upon stimulation, leading to inhibition of furin activity and platelet aggregation [12]. Our findings that serpinB8 is mostly present in monocytes and virtually absent in macrophages suggest a phenotype-driven regulatory step. Even though furin is mostly present in the organelle fraction in macrophages, whereas serpinB8 is predominantly found in the cytoplasm of monocytes, these findings do not exclude serpinB8 as a potential furin-like PCSK inhibitor *per se*. While interaction of furin and MT1-MMP on the cell surface has been described earlier [11,25], the initial maturation step of pro-MT1-MMP occurs most likely within TGN/early endosomes [10,11]. Our immunoblotting data showed that the Golgi-disturbing agent BFA inhibited pro-MT1-MMP maturation, supporting intracellular activation. Since furin-like PCSKs cycle between the TGN and the cell surface via endosomal pathways, serpinB8 inhibiting furin-like PCSKs along this route is likely possible. Accordingly, reexpression of serpinB8 in macrophages inhibited processing of the pro-form of the type I collagenase MT1-MMP comparable to the furin-like PCSK inhibitor dec-CMK, underscoring serpinB8 functioning as a potent furin-inhibitor.

Comparable to the inhibition of furin-like PCSKs, an MMP-inhibitor as well as surpluses of TIMP-2 and TIMP-3 inhibited macrophage migration, whereas the MMP-9 inhibitor TIMP-1 had no effect. This further supports that rather furin-like PCSK dependent activated MT1-MMP is the key regulator in MCP-1-directed migration, since its activity is not significantly affected by TIMP-1 [20]. Substantiating our findings by using ob*/*ob mice as a model of fat tissue inflammation we demonstrate that WAT culture supernatants from ob*/*ob mice containing significant increases in MCP-1 gene expression strongly facilitated macrophage migration compared to lean littermates. This obese WAT-enhanced macrophage migration was inhibited by both furin-like PCSK and MMP inhibition, underlining the concept that a PCSK-driven MMP-activation cascade is important for WAT inflammation.

Among the PCSK family, mutations causing partial deficiency in PCSK1 have been linked to obesity, and mutations in PCSK9 are associated with hypercholesterolemia and coronary heart disease [26,27]. However, little is known about the expression and function of the prototype PCSK, furin, in metabolic diseases. In the present study we show that furin and MT1-MMP are significantly increased in inflammatory WAT from ob*/*ob mice. Moreover, transcript levels of PCSK5, a PCSK with overlapping localization and substrate specificity, was significantly induced, which also might impact on altered convertase activity in WAT. Indeed, whereas furin-specific knockdown significantly inhibited pro-MT1-MMP activity in macrophages, only the general furin-like PCSK inhibitor dec-CMK completely abrogated its activation [10]. This indicates potential PCSK redundancy in pro-MT1-MMP activation, observed earlier by partial MT1-MMP activation in furin-deficient cells [8]. Likewise, using purified PCSKs and soluble MT1-MMP, Remacle et al. [11] found that PCSK5 may substitute for furin in MT1-MMP activation, whereas other PCSKs like PCSK7 were rather insignificant contributors [11]. This is supported by a recent study demonstrating that PCSK7 is distinct from furin in its zymogen activation, subcellular localization, and trafficking [28], thus stressing additional requirements beyond a common di-basic PCSK cleavage motif.

Several studies support the importance of MMPs and PCSKs in adipogenesis [29,30]. MMPs and TIMPs are differentially expressed in adipose tissue in genetic (*db/db* and ob*/*ob mice) and diet-induced obesity models [5]. Thus, whereas upregulation of MMP-2, -3, -12, -19 and MT1-MMP was shown in the stromal-vascular compartment together with TIMP-2, which is required for MMP-2 activation [7], TIMP-3 expression was decreased in obese WAT [5]. Likewise, upon MNC maturation we found downregulation of TIMP-3. Interestingly, this MT1-MMP inhibitor has been shown to characterize an invasive macrophage phenotype within the arterial wall [31] and to protect from metabolic inflammation and insulin resistance *in vivo* when overexpressed in macrophages [32].

Within the MMP superfamily MT1-MMP is unique in its intracellular zymogen activation by furin-like PCSKs, its membrane-bound localization as well as its multiplicity of proteolytic and non-proteolytic properties [7]. Indeed, the importance of MT1-MMP for a continuous remodeling of the adipocytes’ pericellular microenvironment and thus fat cell expansion is highlighted by the finding that adipocyte differentiation is lost and WAT development aborted in knockout mice, resulting in a lipodystrophic phenotype [29]. Comparably, in mice in which the MT1-MMP-inhibitor TIMP-2 is knocked out, increased expression of adipokines (e.g. IL-6, TNF-α), inflammation and macrophage markers (EMR1, MCP-1), as well as MT1-MMP-dependent collagen turnover is found [33]. In contrast, despite the demonstration that MMP-9 serum levels are increased in obesity [34], its deficiency did not impact on adipogenesis in knockout mice on a high-fat diet [35].

The importance of furin-like PCSKs in MT1-MMP activation in cardiometabolic syndrome is further supported by our patients’ data. Here we demonstrate increased MT1-MMP and its cognate convertase furin in monocytes from obese patients compared to normal weight individuals. Circulating monocytes are characterized by a pro-inflammatory/remodeling phenotype in obese patients [34]. In our study, transcript levels of furin correlated with the patients’ BMI, and MT1-MMP correlated with furin. To our knowledge, this suggests for the first time a concordant regulation of furin and MT1-MMP in monocytes in obese patients towards the pro-migratory/pro-inflammatory status characterizing obesity. Furthermore, while furin was increased, levels of serpinB8 were not significantly altered, supporting a disruption in the balance of furin and its inhibitor in adipose patients. Along with furin and MT1-MMP, resistin, an adipokine typically increased in obesity as well as atherosclerosis [21,36], was coordinately upregulated in monocytes from obese patients. Because MCP-1 is the major chemoattractant derived from obese WAT [22], and resistin is abundantly found in circulating MNCs in obesity [37], regulation of furin, serpinB8 and MT1-MMP by these adipokines was compared. Whereas MCP-1 had no impact, resistin strongly induced furin and MT1-MMP in monocytes, with no significant impact on serpinB8. This suggests that resistin may be a contributor to MNC furin/MT1-MMP regulation. Interestingly, regulation of hepatic LDL receptors by resistin involves the related PCSK family member PCSK9, recently identified as a novel target in patients suffering from severe dyslipidemia [38].

In conclusion, this study demonstrates upregulation of furin-like PCSKs (furin and PCSK5) for the first time and, furthermore, an imbalance of furin and its inhibitor serpinB8 in obesity. We show that furin-dependent MMP-activation facilitates WAT-driven macrophage chemotaxis, which is crucial for obesity-associated inflammation. This cascade is induced by resistin and found to be increased in WAT from obese mice as well as in MNCs from obese patients. Dissecting the expression and regulation of furin and its inhibitor serpinB8 will facilitate targeting PCSK-driven inflammation/remodeling in cardiometabolic disorders.

## Supporting Information

Table S1 **Primers used for quantitative real-time PCR.**
(DOC)

Table S2 **Characteristics of wildtype and ob*/*ob mice.**
(DOC)

## References

[1] Berrington de Gonzalez A, Hartge P, Cerhan JR, Flint AJ, Hannan L et al. (2010) Body-mass index and mortality among 1.46 million white adults. N Engl J Med 363: 2211-2219. doi:10.1056/NEJMoa1000367. PubMed: 21121834. 21121834 10.1056/NEJMoa1000367 PMC3066051

[2] Rocha VZ, Libby P (2009) Obesity, inflammation, and atherosclerosis. Nat. Rev Cardiol 6: 399-409. doi:10.1038/nrcardio.2009.55. 10.1038/nrcardio.2009.55 19399028

[3] Weisberg SP, McCann D, Desai M, Rosenbaum M, Leibel RL et al. (2003) Obesity is associated with macrophage accumulation in adipose tissue. J Clin Invest 112: 1796-1808. doi:10.1172/JCI19246. PubMed: 14679176. 14679176 10.1172/JCI19246 PMC296995

[4] Sun K, Kusminski CM, Scherer PE (2011) Adipose tissue remodeling and obesity. J Clin Invest 121: 2094-2101. doi:10.1172/JCI45887. PubMed: 21633177. 21633177 10.1172/JCI45887 PMC3104761

[5] Chavey C, Mari B, Monthouel MN, Bonnafous S, Anglard P et al. (2003) Matrix metalloproteinases are differentially expressed in adipose tissue during obesity and modulate adipocyte differentiation. J Biol Chem 278: 11888-11896. doi:10.1074/jbc.M209196200. PubMed: 12529376. 12529376 10.1074/jbc.M209196200

[6] Chun TH, Inoue M, Morisaki H, Yamanaka I, Miyamoto Y et al. (2010) Genetic link between obesity and MMP14-dependent adipogenic collagen turnover. Diabetes 59: 2484-2494. doi:10.2337/db10-0073. PubMed: 20660624. 20660624 10.2337/db10-0073 PMC3279534

[7] Strongin AY (2010) Proteolytic and non-proteolytic roles of membrane type-1 matrix metalloproteinase in malignancy. Biochim Biophys Acta 1803: 133-141. doi:10.1016/j.bbamcr.2009.04.009. PubMed: 19406172. 19406172 10.1016/j.bbamcr.2009.04.009 PMC2823998

[8] Yana I, Weiss SJ (2000) Regulation of membrane type-1 matrix metalloproteinase activation by proprotein convertases. Mol Biol Cell 11: 2387-2401. doi:10.1091/mbc.11.7.2387. PubMed: 10888676. 10888676 10.1091/mbc.11.7.2387 PMC14927

[9] Seidah NG, Prat A (2012) The biology and therapeutic targeting of the proprotein convertases. Nat Rev Drug Discov 11: 367-383. doi:10.1038/nrd3699. PubMed: 22679642. 22679642 10.1038/nrd3699

[10] Stawowy P, Meyborg H, Stibenz D, Borges Pereira Stawowy N, Roser M, et al. (2005) Furin-like proprotein convertases are central regulators of the membrane type matrix metalloproteinase-pro-matrix metalloproteinase-2 proteolytic cascade in atherosclerosis. Circulation 111: 2820-2827. doi:10.1161/CIRCULATIONAHA.104.502617. PubMed: 15911696. 15911696 10.1161/CIRCULATIONAHA.104.502617

[11] Remacle AG, Rozanov DV, Fugere M, Day R, Strongin AY (2006) Furin regulates the intracellular activation and the uptake rate of cell surface-associated MT1-MMP. Oncogene 25: 5648-5655. doi:10.1038/sj.onc.1209572. PubMed: 16636666. 16636666 10.1038/sj.onc.1209572

[12] Leblond J, Laprise MH, Gaudreau S, Grondin F, Kisiel W et al. (2006) The serpin proteinase inhibitor 8: an endogenous furin inhibitor released from human platelets. Thromb Haemost 95: 243-252. PubMed: 16493485. 16493485 10.1160/TH05-08-0561

[13] Dahlen JR, Jean F, Thomas G, Foster DC, Kisiel W (1998) Inhibition of soluble recombinant furin by human proteinase inhibitor 8. J Biol Chem 273: 1851-1854. doi:10.1074/jbc.273.4.1851. PubMed: 9442015. 9442015 10.1074/jbc.273.4.1851

[14] Silverman GA, Bird PI, Carrell RW, Church FC, Coughlin PB et al. (2001) The serpins are an expanding superfamily of structurally similar but functionally diverse proteins. Evolution, mechanism of inhibition, novel functions, and a revised nomenclature. J Biol Chem 276: 33293-33296. doi:10.1074/jbc.R100016200. PubMed: 11435447. 11435447 10.1074/jbc.R100016200

[15] Strik MC, Bladergroen BA, Wouters D, Kisiel W, Hooijberg JH et al. (2002) Distribution of the human intracellular serpin protease inhibitor 8 in human tissues. J Histochem Cytochem 50: 1443-1454. doi:10.1177/002215540205001103. PubMed: 12417609. 12417609 10.1177/002215540205001103

[16] Ito A, Suganami T, Yamauchi A, Degawa-Yamauchi M, Tanaka M et al. (2008) Role of CC chemokine receptor 2 in bone marrow cells in the recruitment of macrophages into obese adipose tissue. J Biol Chem 283: 35715-35723. doi:10.1074/jbc.M804220200. PubMed: 18977759. 18977759 10.1074/jbc.M804220200

[17] Hallenberger S, Bosch V, Angliker H, Shaw E, Klenk HD et al. (1992) Inhibition of furin-mediated cleavage activation of HIV-1 glycoprotein gp160. Nature 360: 358-361. doi:10.1038/360358a0. PubMed: 1360148. 1360148 10.1038/360358a0

[18] Wood SA, Park JE, Brown WJ (1991) Brefeldin A causes a microtubule-mediated fusion of the trans-Golgi network and early endosomes. Cell 67: 591-600. doi:10.1016/0092-8674(91)90533-5. PubMed: 1657400. 1657400 10.1016/0092-8674(91)90533-5

[19] Ladinsky MS, Howell KE (1992) The trans-Golgi network can be dissected structurally and functionally from the cisternae of the Golgi complex by brefeldin A. Eur J Cell Biol 59: 92-105. PubMed: 1468449. 1468449

[20] Brew K, Nagase H (2010) The tissue inhibitors of metalloproteinases (TIMPs): an ancient family with structural and functional diversity. Biochim Biophys Acta 1803: 55-71. doi:10.1016/j.bbamcr.2010.01.003. PubMed: 20080133. 20080133 10.1016/j.bbamcr.2010.01.003 PMC2853873

[21] Steppan CM, Bailey ST, Bhat S, Brown EJ, Banerjee RR et al. (2001) The hormone resistin links obesity to diabetes. Nature 409: 307-312. doi:10.1038/35053000. PubMed: 11201732. 11201732 10.1038/35053000

[22] Kanda H, Tateya S, Tamori Y, Kotani K, Hiasa K et al. (2006) MCP-1 contributes to macrophage infiltration into adipose tissue, insulin resistance, and hepatic steatosis in obesity. J Clin Invest 116: 1494-1505. doi:10.1172/JCI26498. PubMed: 16691291. 16691291 10.1172/JCI26498 PMC1459069

[23] Gettins PG, Olson ST (2009) Exosite determinants of serpin specificity. J Biol Chem 284: 20441-20445. doi:10.1074/jbc.R800064200. PubMed: 19401470. 19401470 10.1074/jbc.R800064200 PMC2742806

[24] Gillard A, Scarff K, Loveland KL, Ricardo SD, Bird PI (2006) Modulation and redistribution of proteinase inhibitor 8 (Serpinb8) during kidney regeneration. Am J Nephrol 26: 34-42. doi:10.1159/000091784. PubMed: 16508245. 16508245 10.1159/000091784

[25] Boucher E, Mayer G, Londono I, Bendayan M (2006) Expression and localization of MT1-MMP and furin in the glomerular wall of short- and long-term diabetic rats. Kidney Int 69: 1570-1577. doi:10.1038/sj.ki.5000316. PubMed: 16541018. 16541018 10.1038/sj.ki.5000316

[26] Cohen JC, Boerwinkle E, Mosley TH Jr., Hobbs HH (2006) Sequence variations in PCSK9, low LDL, and protection against coronary heart disease. N Engl J Med 354: 1264-1272. doi:10.1056/NEJMoa054013. PubMed: 16554528. 16554528 10.1056/NEJMoa054013

[27] Creemers JW, Choquet H, Stijnen P, Vatin V, Pigeyre M et al. (2012) Heterozygous mutations causing partial prohormone convertase 1 deficiency contribute to human obesity. Diabetes 61: 383-390. doi:10.2337/db11-0305. PubMed: 22210313. 22210313 10.2337/db11-0305 PMC3266396

[28] Rousselet E, Benjannet S, Hamelin J, Canuel M, Seidah NG (2011) The proprotein convertase PC7: unique zymogen activation and trafficking pathways. J Biol Chem 286: 2728-2738. doi:10.1074/jbc.M110.192344. PubMed: 21075846. 21075846 10.1074/jbc.M110.192344 PMC3024769

[29] Chun TH, Hotary KB, Sabeh F, Saltiel AR, Allen ED et al. (2006) A pericellular collagenase directs the 3-dimensional development of white adipose tissue. Cell 125: 577-591. doi:10.1016/j.cell.2006.02.050. PubMed: 16678100. 16678100 10.1016/j.cell.2006.02.050

[30] Croissandeau G, Basak A, Seidah NG, Chrétien M, Mbikay M (2002) Proprotein convertases are important mediators of the adipocyte differentiation of mouse 3T3-L1 cells. J Cell Sci 115: 1203-1211. PubMed: 11884519. 11884519 10.1242/jcs.115.6.1203

[31] Johnson JL, Sala-Newby GB, Ismail Y, Aguilera CM, Newby AC (2008) Low tissue inhibitor of metalloproteinases 3 and high matrix metalloproteinase 14 levels defines a subpopulation of highly invasive foam-cell macrophages. Arterioscler Thromb Vasc Biol 28: 1647-1653. doi:10.1161/ATVBAHA.108.170548. PubMed: 18566294. 18566294 10.1161/ATVBAHA.108.170548 PMC2851011

[32] Menghini R, Casagrande V, Menini S, Marino A, Marzano V et al. (2012) TIMP3 overexpression in macrophages protects from insulin resistance, adipose inflammation, and nonalcoholic fatty liver disease in mice. Diabetes 61: 454-462. doi:10.2337/db11-0613. PubMed: 22228717. 22228717 10.2337/db11-0613 PMC3266402

[33] Jaworski DM, Sideleva O, Stradecki HM, Langlois GD, Habibovic A et al. (2011) Sexually dimorphic diet-induced insulin resistance in obese tissue inhibitor of metalloproteinase-2 (TIMP-2)-deficient mice. Endocrinology 152: 1300-1313. doi:10.1210/en.2010-1029. PubMed: 21285317. 21285317 10.1210/en.2010-1029 PMC3060627

[34] Ghanim H, Aljada A, Hofmeyer D, Syed T, Mohanty P et al. (2004) Circulating mononuclear cells in the obese are in a proinflammatory state. Circulation 110: 1564-1571. doi:10.1161/01.CIR.0000142055.53122.FA. PubMed: 15364812. 15364812 10.1161/01.CIR.0000142055.53122.FA

[35] Van Hul M, Piccard H, Lijnen HR (2010) Gelatinase B (MMP-9) deficiency does not affect murine adipose tissue development. Thromb Haemost 104: 165-171. doi:10.1160/TH09-10-0739. PubMed: 20431850. 20431850 10.1160/TH09-10-0739

[36] Reilly MP, Lehrke M, Wolfe ML, Rohatgi A, Lazar MA et al. (2005) Resistin is an inflammatory marker of atherosclerosis in humans. Circulation 111: 932-939. doi:10.1161/01.CIR.0000155620.10387.43. PubMed: 15710760. 15710760 10.1161/01.CIR.0000155620.10387.43

[37] Savage DB, Sewter CP, Klenk ES, Segal DG, Vidal-Puig A et al. (2001) Resistin / Fizz3 expression in relation to obesity and peroxisome proliferator-activated receptor-gamma action in humans. Diabetes 50: 2199-2202. doi:10.2337/diabetes.50.10.2199. PubMed: 11574398. 11574398 10.2337/diabetes.50.10.2199

[38] Roth EM, McKenney JM, Hanotin C, Asset G, Stein EA (2012) Atorvastatin with or without an Antibody to PCSK9 in Primary Hypercholesterolemia. N Engl J Med 367: 1891-1900. doi:10.1056/NEJMoa1201832. PubMed: 23113833. 23113833 10.1056/NEJMoa1201832

