# Supplementary material for: Proprotein Convertase Subtilisin/Kexin Type 3 Promotes Adipose Tissue-Driven Macrophage Chemotaxis and Is Increased in Obesity
Source: PLoS One. 2013 Aug 6;8(8):e70542. doi: 10.1371/journal.pone.0070542 (PMC3735592; doi:10.1371/journal.pone.0070542)
Supplement: Table S1 — Primers used for quantitative real-time PCR. (DOC) [file pone.0070542.s001.doc]

**Supporting Table S1**

**Primers used for quantitative real-time PCR**

**A. Human Primers**

| **Primer** | **Forward (100nmol/L)** | **Reverse (100nmol/L)** |
| --- | --- | --- |
| 18S | CGGCTACATCCAAGGAA | GCTGGAATTACCGCGGT |
| Furin | TATGGCTACGGGCTTTTGGA | GCCGTTTCCCGATGTCTTT |
| MCP-1 | GCCTCCAGCATGAAAGTCTC | CATTGATTGCATCTGGCTGA |
| MT1-MMP | GCCTGCGTCCATCAACACT | ACACCCAATGCTTGTCTCCTTT |
| Resistin | ATAAGCAGCATTGGCCTGGA | CTGGCAGTGACATGTGGTCT |
| SERPINB8 | TGGCAGAGAAGACTGAAGGTAAGA | ATTCACAAGGACCAGCTTTGTCA |
| Vimentin | TGCCCTTAAAGGAACCAATGA | GCTTCAACGGCAAAGTTCTCTT |

**B. Mouse Primers**

| **Primer** | **Forward (100nmol/L)** | **Reverse (100nmol/L)** |
| --- | --- | --- |
| 18S | GGACTCTTTCGAGGCCCTGTA | CACCAGACTTGCCCTCCAAT |
| CD68 | GGACTACATGGCGGTGGAATA | GATGAATTCTGCGCCATGAA |
| F4/80 | TTTCCTCGCCTGCTTCTTC | CCCCGTCTCTGTATTCAACC |
| Furin | GGCTTTCATGACAACCCATT | GGCTGGATGTGAGGGTCTT |
| PCSK5 | TGGGCCAGGATTCAAGAACT | TGCCCAGGACTCTTCCGTA |
| MCP-1 | AGCACCAGCCAACTCTCACT | CGTTAACTGCATCTGGCTGA |
| MT1-MMP | TGCCTACGAGAGGAAGGATG | CGTCAAACACCCAGTGCTTA |
| Resistin | CAGAAGGCACAGCAGTCTTG | GGGCTGCTGTCCAGTCTATC |
| SERPINB8 | AAGGGTGCAGGAAACACATC | GGCATGCCTCTCGTGTACTT |
