# Supplementary material for: Proprotein Convertase Subtilisin/Kexin Type 3 Promotes Adipose Tissue-Driven Macrophage Chemotaxis and Is Increased in Obesity
Source: PLoS One. 2013 Aug 6;8(8):e70542. doi: 10.1371/journal.pone.0070542 (PMC3735592; doi:10.1371/journal.pone.0070542)
Supplement: Table S2 — Characteristics of wildtype and ob/ob mice. (DOC) [file pone.0070542.s002.doc]

**Supporting Table S2**

**Characteristics of wildtype and *ob/ob* mice (mean ± S.D.)**

| **Characteristics/Parameter** | **wildtype mice (*n*=)** | ***ob/ob* mice (*n*=)** | ***P* for difference** |
| --- | --- | --- | --- |
| Body weight, *g* | 28.38±2.24 (*5*) | 50.13±0.75 (*5*) | <0.001 |
| Gondal fat tissue, *g* | 0.26±0.10 (*5*) | 3.70±0.25 (*5*) | <0.001 |
| Sucutaneous fat tissue, *g* | 0.16±0.09 (*5*) | 4.32±0.35 (*5*) | <0.001 |
| Liver, *g* | 1.26±0.17 (*5*) | 3.34±0.26 (*5*) | <0.001 |
| Kidneys, *g* | 0.35±0.05 (*5*) | 0.37±0.02 (*5*) | n.s. |
| Left ventricle, *g* | 0.18±0.05 (*4*) | 0.13±0.01 (*4*) | <0.01 |
